# Aerobic exercise and gut flora: a key link to improved cognitive impairment in mice with Parkinson’s disease

**DOI:** 10.3389/fnagi.2025.1630003

**Published:** 2025-10-21

**Authors:** Wen Peng Shan, Shi Lei Yan, Yuan Yuan Guo, Hua Ke Yang, Jing Chun Wang, Jie Xiang

**Affiliations:** ^1^Public Experiment Platform, Xuzhou Medical University, Xuzhou, China; ^2^Department of Rehabilitation Medicine, Affiliated Hospital of Xuzhou Medical University, Xuzhou, China

**Keywords:** Parkinson’s disease, aerobic exercise, gut flora, cognitive impairment, FNDC5

## Abstract

**Background:**

Parkinson’s disease (PD) is a common neurodegenerative disorder. It is marked by motor dysfunction and cognitive decline. In recent years, scientific studies have found that PD’s pathogenesis may be tied to an imbalance in the gut microbiota. This offers new perspectives for PD treatment. Modulating the gut microbiota is recognized as a potential way to enhance PD symptoms. While aerobic exercise can positively influence the gut microbiota, research on how the gut microbiota mediates aerobic exercise’s effects on PD cognitive impairment is still limited. Thus, this study aimed to explore the potential mechanisms by which aerobic exercise improves cognitive impairment in PD patients. It does so by modulating the gut microbiota’s structure and, in turn, improving cognitive function. Through this study, we hope to offer new strategies and a theoretical basis for treating PD cognitive impairment.

**Methods:**

This study focused on the potential neuroprotective effects of long-term aerobic exercise in an MPTP-induced PD mouse model. Research methods included using 16S rRNA gene sequencing and plasma untargeted metabolomics to precisely describe the composition of the mouse gut microbiota and its metabolite changes. We also monitored the mice’s motor and cognitive functions via behavioral assessments. Pathological features and molecular-level changes in PD mice were analyzed using morphological and molecular biology techniques. To further study the role of gut microbes in aerobic exercise, we conducted antibiotic treatment experiments on mice. Finally, Pearson correlation analysis was used to explore the correlation between gut microbiota, plasma metabolite outcomes, and molecules related to cognitive function.

**Results:**

Our results showed that aerobic exercise effectively intervened in PD mice. It alleviated PD-related pathological impairments and cognitive deficits and promoted the secretion of FNDC5 and BDNF, producing neuroprotective effects. Aerobic exercise regulated the gut flora imbalance in PD mice. 16S rRNA analysis revealed a significant increase in the abundance of *Alloprevotella, Akkermansia, Lachnospiraceae_NK4A136_group, Bacteroides,* and *Prevotellaceae_UCG-001*. In contrast, the abundance of *Parabacteroides, Helicobacter, Alistipes,* and *Odaribacter* decreased significantly. The gut flora mediated the role of aerobic exercise by regulating FNDC5 secretion through PGC1-*α*/CREB and influencing BDNF production.

**Conclusion:**

Aerobic exercise improves gut flora imbalance in PD mice. It also attenuates PD-related pathological impairments and cognitive deficits. However, its efficacy on non-motor symptoms can be nullified by antibiotics. The gut flora-mediated aerobic exercise exerts neuroprotective effects on PD by regulating FNDC5 secretion via PGC1-*α*/CREB.

## 1 Introduction

Parkinson’s disease (PD) is the second most common neurodegenerative disorder globally, after Alzheimer’s disease (Song et al., 2022). PD patients typically have resting tremor, muscle rigidity, bradykinesia, and postural and balance problems (Váradi, 2020; Öksüz et al., 2022). Moreover, they often experience non-motor symptoms like cognitive deficits, constipation (Jones et al., 2020), and autonomic dysfunction (Iranzo et al., 2013; Ferraris et al., 2009). Pathologically, two core features of PD are the progressive loss of dopaminergic neurons in the substantia nigra pars compacta (Zhou et al., 2023; Coukos and Krainc, 2024) and the abnormal accumulation of *α*-synuclein (Vázquez-Vélez and Zoghbi, 2021; Matsui and Takahashi, 2024). Although PD’s exact pathogenesis remains unclear, it’s widely believed to result from a mix of genetic and environmental factors, following the “double whammy” model (Kou Li et al., 2018; Flønes et al., 2022). Currently, PD treatment mainly relies on pharmacological methods such as dopamine replacement therapy and surgical procedures like deep brain stimulation (Stocchi et al., 2024). However, due to limited knowledge of PD pathogenesis, existing treatments can mainly relieve motor symptoms (Gonzalez-Latapi et al., 2020). They are ineffective for non-motor symptoms like cognitive impairment (Jing et al., 2023) and cannot effectively stop disease progression. Thus, in-depth study of PD pathogenesis and development of new therapeutic strategies are crucial for improving PD patients’ quality of life and slowing disease progression. In PD, non-motor symptoms often come before motor symptoms (Kaiserova et al., 2021). Research suggests PD may start in the gastrointestinal tract (Beach et al., 2016). Gut microorganisms play a key role in PD progression there. They do so by regulating metabolites, maintaining the integrity of the gastrointestinal epithelial barrier, and modulating immune function (Suresh et al., 2024; Castro-Vidal et al., 2024). Gastrointestinal dysfunction precedes motor symptoms in PD, which is also seen in clinical cases (Safarpour et al., 2024; Bissacco et al., 2024). Braak et al. proposed that PD may begin in the olfactory and vagus nerves of the intestinal tract (Bhattarai et al., 2021). Misfolded alpha-synuclein spreads from the periphery to the central nervous system (Hawkes et al., 2010). This abnormal protein folding links to gut flora imbalance, common in PD patients (Bissacco et al., 2024). PD patients with gastrointestinal symptoms have a different gut microbial composition compared to healthy people (Beach et al., 2016). There are significant differences in the alpha and/or beta diversity of the gut microbial community in PD patients, especially at the genus level (Barichella et al., 2019; Qian et al., 2018). For example, the genera *Lactobacillaceae, Christensenellaceae,* and *Roseburia* spp. increase, while *Prevotellaceae* and *Lachnospiraceae* decrease. The current study hypothesizes that the gastrointestinal tract may contribute to PD development by changing the intestinal flora composition. The bidirectional interactions between the peripheral and central nervous systems, forming the microbiota-gut-brain axis (MGBA) (Chapelet et al., 2019; Dogra et al., 2022), further drive disease progression and the appearance of motor symptoms. This implies that gut flora can affect host health. So, improving gut flora in PD patients could offer a new treatment strategy for PD and its associated cognitive dysfunction (Jones et al., 2020).

Aerobic exercise is a crucial part of exercise rehabilitation for PD patients. It has been proven effective in multiple ways. It can improve motor symptoms in PD patients (Marusiak et al., 2019; van der Kolk et al., 2019). Also, it helps reduce anxiety and depression symptoms (Costa et al., 2024). Besides, it increases bowel movements, thus improving non-motor symptoms like constipation (Wang et al., 2023). Current research shows that aerobic exercise works in two main ways. It increases dopamine release and activates neurotrophic factors (Solinas et al., 2019). Moreover, it may positively impact non-motor symptoms of PD by promoting the production of the FNDC5 protein (Leger et al., 2024; Yao et al., 2024). In healthy populations, aerobic exercise can significantly modulate the gut microbiota and metabolic status (Varghese et al., 2024). This affects gut microecological homeostasis. However, its effect on the gut microbiota composition in PD patients is still unknown. Also, we do not know if the gut flora affects cognitive deficits in PD patients by promoting FNDC5 production. To study this, we used a 1-methyl-4-phenyl-1,2,3,6-tetrahydropyridine (MPTP)-induced mouse model of PD. This model can simulate the progressive loss of dopaminergic neurons and the misfolding and stacking of *α*-ynuclein. We modulated the intestinal flora with aerobic exercise and antibiotics. Then, we observed changes in cognitive function and the improvement of PD-related pathological features in mice. Our study has two main findings. First, it identified the importance of aerobic exercise in remodeling the gut flora of PD patients. Second, it verified the relationship between the FNDC5 molecule, which is closely related to altered cognitive function, and changes in gut flora. This provides a theoretical basis for promoting PD rehabilitation from the microbe-gut-brain axis perspective.

## 2 Methodologies

### 2.1 Animals and handling

We used male C57BL/6 J mice from the Animal Experiment Center of Xuzhou Medical University. They were 6–8 weeks old and weighed 20–30 g. The mice were kept in strictly controlled conditions. The temperature was 23 ± 2 °C, the humidity was 45 ± 5%, and there was a 12-h light and 12-h dark cycle. All experimental operations were approved by the Ethics Committee of Xuzhou Medical University. We followed the Guidelines for the Care and Use of Laboratory Animals issued by the National Institutes of Health (Decree No. 55 of the Ministry of Health, Revised Edition, 1998).

Before the experiment, we freshly prepared all reagents and drugs. Based on a previous study (Park et al., 2024), we made a PD mouse model with MPTP. First, we diluted MPTP (Cat#HY-15608, MedChemExpress, USA) to 3 mg/mL with sterile saline. At the same time, we made a 10 mg/mL solution of probenecid (Cat#P129440, Aladdin, China). Then, we injected mice intraperitoneally every 3.5 days. The dose was 30 mg/kg of MPTP and 250 mg/kg of probenecid, for a total of 10 times. Control mice got an equal amount of saline. MPTP-injected Parkinsonian mice were randomly allocated into two groups (*n* = 12 per group): one group underwent aerobic exercise training, while the other remained sedentary. Concurrently, saline-injected control mice (*n* = 12) similarly remained sedentary as the control group.

For aerobic exercise training, we first let the mice in the aerobic training group get used to the running platform for a week. This reduced their stress response (Su et al., 2024). Then, they started formal training. The treadmill speed was 12 m/min. Each session had two 30-min runs with a 5-min rest between them, as in previous studies (Zhou et al., 2022). Control mice and PD mice had a 65-min simulated training on a treadmill at 0 m/min. The whole training program was 5 days a week for 10 weeks.

In the antibiotic treatment experiments, we used a method like previous studies (Sampson et al., 2016). We prepared ampicillin 1 g/L (Cat#MB1507-2, meilunbio, China), neomycin 1 g/L (Cat#MB1716-2, meilunbio, China), metronidazole 1 g/L (Cat#MB2200-1, meilunbio, China), and vancomycin hydrochloride 0.5 g/L (Cat#MB1260-4, meilunbio, China) in solution. We quickly added them to sterile water. After five-week antibiotic treatment, we removed about 99% of the intestinal flora. Mice were administered an antibiotic cocktail via continuous ad libitum feeding for 5 weeks, while the non-antibiotic-treated group received autoclaved drinking water as control.

### 2.2 Behavioral tests

To ensure statistical independence across all data samples, each mouse in our experimental design underwent only a single test per experimental paradigm.

#### 2.2.1 Morris water maze test

We adopted the methodology of a previous study (Seo et al., 2023) and used the Morris water maze test to evaluate cognitive function and learning efficiency in mice. The maze was divided into target quadrant by ANYMaze software (MED Associates, Georgia, VT, United States). The fifth quadrant was inside the fourth quadrant and had a movable circular escape platform.

The experiment had three phases: acclimatization, continuous training, and evaluation. In the acclimatization phase, the maze was filled with water, and the platform was 1 cm above the water surface. Mice were put into the maze from a random quadrant. If they found the platform within 60 s, they remained on the platform for 60 s. If not, the experimenter guided them to the platform, and they stayed for 15 s.

In the training phase, the water level was set 1 cm above the platform, and non-toxic white ink was added to opacify the water, making it harder to find the platform. Mice were placed in 4 random quadrants daily to find the platform.

In the evaluation phase, we kept the same test conditions and put the mice in the second quadrant to find the platform. We used a computer to track the mice’s paths, calculate their average and peak speeds, and record the time they took to find the platform (latency) and the number of times they entered the target quadrant (previously quadrant 4).

#### 2.2.2 Open field experiments

The open field experiment aimed to carefully assess the mice’s instinctive behavioral responses to new environments, including exploration, anxiety-like behavior, activity level, and emotional states like anxiety and depression. To reduce the impact of unfamiliar environment on the results, mice were given half an hour to get used to the experimental environment before the experiment started.

During the experiment, the mice were randomly placed in a large test chamber to freely explore for 5 min. The ANY-maze video tracking system used a video camera to accurately record every movement of the mice. This data was used for later behavioral pattern analysis.

#### 2.2.3 Rotating rod experiment

We used the rotarod test to measure motor coordination in mice. Based on a previous study (Cui et al., 2022), mice were placed on a rotating bar that accelerated gradually from 4 to 40 rpm in 5 min. We recorded the time it took for them to fall (latency). To make the test accurate, all mice had a 5 -day pre-training phase before the formal test. Through three independent experiments, we calculated the mean latency to fall(s) of the mice as a measure of their motor function.

#### 2.2.4 New object recognition experiments

Based on previous research (Li et al., 2012), the experiment started with mice acclimatizing in a 40 × 40 × 40 cm square open field. The next day, two identical objects were placed in different locations in the field. Then, the mice were returned to their home cages and had a memory retention period of either 0.5 h or 24 h. During this time, one of the objects was replaced with a new one for a new object recognition test. At the end of the memory retention period, the mice were put back into the open field for a 5-min observation of their exploratory behavior. Effective exploration meant the mice focused their attention within 1 cm of the object. Non-exploratory behaviors (e.g., turning, climbing) was considered ineffective exploration. We used the discrimination index DI = (T_new-T_familiar)/(T_new+T_familiar) × 100% to measure the difference in exploration time between familiar and unfamiliar objects for the mice. The ANY-maze software automatically tracked and recorded the entire experimental process to record the mice’s activity trajectories.

### 2.3 Western blot (WB)

We sacrificed the mice and collected tissues, which were then frozen at −80 °C for storage. We lysed the tissues with RIPA Lysate (Cat#WB3100, New Cell & Molecular Biotech, SZ, CHN) and centrifuged them at 12,000 rpm for 20 min at 4 °C to get the total protein in the supernatant. We determined the protein concentration using the BCA Protein Assay Kit (Cat#P0010S, Beyotime, SH, CHN). We added Sodium dodecyl sulfate SDS (Cat#VP6006, Vicmed, XZ, CHN) to the loading buffer and boiled it at 95 °C for 10 min to denature the protein. We loaded an appropriate amount of protein onto a 10% SDS-PAGE (Cat#P2011, P2012, P2013, NCM, SZ, CHN) for electrophoresis and transferred the protein to a 0.45 μm PVDF (Cat#VFISEQ00010, VFPVH00010, Vicmed, XZ, CHN) membrane. We blocked the membranes for 30 min with Rapid Closure Solution (Cat#P30500, NCM, SZ, CHN). The membranes were incubated with the primary antibody overnight at 4 °C and with the fluorescent enzyme-labeled secondary antibody for 1 h at room temperature. We developed the final image using a dual infrared laser imaging system (Cat#Odyssey CLX, Licor, USA) and analyzed it with imageJ1.49v. The following antibodies were used: anti-TH antibody (Cat#SAB2701683, Sigma, USA), anti-ZO-1 antibody (Cat#13663, CST, USA), anti-Occludin antibody (Cat#ab222691, Abcam, UK), anti-cluadin-5 antibody (Cat#ab172968, Abcam, UK), anti-BDNF antibody (Cat# 28205-1-AP, Proteintech, USA), anti-FNDC5 antibody (Cat# 82671-1-RR, Proteintech, USA), anti-cAMP antibody (Cat# 82940-1-RR, Proteintech, USA) anti-PGC1-*α* (Cat# HY-P80783, MedChemExpress, USA) antibody, anti-CREB antibody (Cat# HY-P80092, MedChemExpress, USA), anti-*β*-actin antibody (Cat# 60008-1-Ig, Proteintech, USA), Anti-GAPDH Antibody (Cat# 10494-1-AP, Proteintech, USA), Goat Anti-Rabbit Antibody (Cat# VSA27, Vicmed, XZ, CHN), Goat Anti-Mouse Antibody (Cat# VSA32, Vicmed, XZ, CHN).

### 2.4 Immunofluorescence

We sacrificed the mice and collected their brains, which were fixed in 4% paraformaldehyde for 48 h, with the solution changed every 24 h. On the third day, we performed dehydration with a 15% sucrose solution, and on the fourth day, with a 30% sucrose solution. When dehydration was complete, we fixed the sections on a cryostat freezing stage using an embedding agent and collected coronal sections 20-μm-thick coronal sections. The section area included the hippocampus and substantia nigra, and each slide had three sections, with three sections used for simultaneous analysis. We washed the embedding agent in PBS solution at room temperature, blocked the sections with 10% goat serum for 90 min at room temperature, incubated the primary antibody at 4 °C overnight, and then incubated with a fluorescent enzyme-labeled secondary antibody for fluorescence microscopy by incubating for 2 h at room temperature using a DAPI-containing blocking solution (Cat# P0131, Beyotime, SH, CHN). The following antibodies were used: anti-TH antibody (Cat#AB152, Sigma, USA), murine anti-TH coupled Alexa Fluor 488 antibody (Cat# MAB318-AF488, Sigma, USA).

### 2.5 Paraffin sections and HE staining

Before sampling, we perfused the mouse tissues sequentially with 20 mL of saline and 20 mL of 4% paraformaldehyde. Then, we removed the colon tissues and immersed them in a 4% paraformaldehyde solution stored at 4 °C overnight. After washing with water, we dehydrated the tissues sequentially: 75% ethanol for 1–2 h, 80% ethanol for 1–2 h, 90% ethanol for 1 h, 95% ethanol for 0.5 h, and anhydrous ethanol for 20 min twice. We used xylene to clear for 20 min for paraffin embedding and sectioning, and 3 sections per slide were used for triplicate analyses. After making the paraffin sections, we put the paraffin sections sequentially into xylene I for 10 min, xylene II for 10 min, xylene III for 10 min, anhydrous ethanol I for 5 min, anhydrous ethanol II for 5 min, 90% alcohol for 5 min, 80% alcohol for 5 min, 70% alcohol for 5 min, and 50% alcohol for 5 min for gradient dewaxing. We stained the paraffin sections with hematoxylin for 0.5–1 min, differentiated them with 1% hydrochloric acid alcohol for several seconds, returned them to blue with 1% ammonia solution for 1 min, stained them with eosin for several seconds, then immersed the paraffin sections successively in 75% ethanol and 85% ethanol for 2 min each, twice in anhydrous ethanol for 5 min, cleared them with xylene for 5 min, and sealed the sections with neutral gum. We observed the sections in bright field under a microscope and scored them histologically. 3.6 Golgi-Cox staining.

After necropsy, we perfused the mice with saline and 4% paraformaldehyde, took the whole brain, and prepared solutions A, B, C, D, E according to the staining kit (Cat#FD Rapid GolgiStain Kit, FD NeuroTechnologies, USA). Among them, 2 mL of liquid A and 2 mL of liquid B were made into AB, and 3 mL of liquid D and 3 mL of liquid E were made into DE. We first immersed the whole brain in AB liquid for 24 h.and then replaced it with freshly made AB liquid for 13 h. One day before slicing, we prepared chromium potassium sulfate-gelatin slides in the ratio of 1 g gelatin, 0.1 g chromium potassium sulfate, and 200 mL ddH₂O. We attached 100 μm brain slices to the glued slides, then dripped liquid C and dried them at room temperature for 1–3 days. After that, we rinsed the slices in PBS 3 times, then dripped DE solution for 10 min. We dehydrated the slices in a gradient of 50, 75, and 95% alcohol for 4 min each, then with 100% alcohol for 4 min, 4 times, and finally with xylene for 4 min, 3 times, and sealed them with transparent gum. We observed and photographed the slices with a digital section scanning system and counted the dendritic spine density of neurons in the CA1 region of the mouse hippocampus in each group according to the formula dendritic spine density = spine count (number)/dendrite length (μm). The whole process was carried out under dark conditions, except for the sampling and perfusion.

### 2.6 Amplicon

We collected fecal samples and stored them in an −80 °C refrigerator. Subsequently, we entrusted these samples to Beijing Novozymes. Here are the detailed procedures: Fecal genomic DNA was extracted via the magnetic bead method. After subjecting it to 1% agarose gel electrophoresis, we diluted the DNA to a concentration of 1 ng/μL to assess its purity and concentration. Specific primers were employed to amplify target regions. The 16S V4 region (515F/806R) was used to analyze bacterial diversity, the 18S V4 region (528F/706R) for eukaryotic microbial diversity, and the ITS1 region (ITS5-1737F/ITS2-2043R) for fungal diversity. Additionally, the amplification covered 16S V3-V4/V4-V5/V5-V7, Archaeal 16S V4–V5/V8 regions, 18S V9, and ITS2 regions. The PCR reaction system contained 15 μL of Phusion® High-Fidelity PCR Master Mix, 0.2 μM of primers, and 10 ng of DNA template. The reaction conditions included pre-denaturation at 98 °C for 1 min, followed by 30 cycles (98 °C for 10s, 50 °C for 30s, 72 °C for 30s), and a final extension at 72 °C for 5 min. Post-purification, the library was constructed using the NEB Next® Ultra™ II FS DNA PCR-free Library Prep Kit. After passing Qubit and Q-PCR quantification, PE 250 sequencing was carried out on the NovaSeq 6000 platform.

### 2.7 Plasma untargeted metabolomics assays

We collected plasma samples and stored them in an −80 °C refrigerator. These samples were then sent to Beijing Novozymes for analysis. Here’s how the process worked: First, we added 100 μL of each sample to 400 μL of an 80% methanol aqueous solution. We then vortexed and shook the mixture to extract the supernatant. Next, we diluted this supernatant to a 53% methanol solution. After that, we centrifuged it again and collected the supernatant for LC-MS analysis.

For quality control (QC), we took a medium-volume aliquot from each experimental sample and mixed them together to create the QC samples. As for blank samples, we used a 53% methanol aqueous solution instead.

Once we identified the metabolites, we annotated them using three databases: the KEGG database,^1^ the HMDB database,^2^ and the LIPIDMaps database.^3^ We used the R package Pheatmap to plot cluster heatmaps. Also, we normalized the metabolite data using the z-score. To analyze the relationships between different metabolites, we used the R language. Specifically, we calculated the Pearson’s correlation coefficient with the cor() function and determined statistical significance with cor.mtest() in R. A *p*-value <0.05 was considered statistically significant.

### 2.8 Image processing

We analyzed the HE (hematoxylin and eosin) image data. We observed the samples in the bright field under a microscope and scored them histologically. For inflammatory infiltration, we assigned scores as follows: 0 for normal (no increase in inflammatory cells); 1 when there was an increased number of inflammatory cells in the lamina propria; 2 if the inflammatory cells extended confluently into the submucosa; and 3 for dense inflammatory infiltration.

Regarding tissue damage, a score of 0 meant no mucosal damage. A score of 1 indicated lymphoepitheliopathy. A score of 2 was given for surface mucosal erosion or focal ulceration. And a score of 3 was assigned when there was extensive mucosal damage that extended into the deeper structures of the intestinal wall. The total histological score for each sample was the sum of these two scores. 3.10 Data Processing.

In this study, we used GraphPad Prism 9.0 software to analyze all the data. For data that followed a normal distribution and had uniform variance, we used one-way analysis of variance (ANOVA) to compare data among multiple groups. We expressed the experimental data as the mean ± standard error of the mean (±SEM). A *p*-value <0.05 was considered statistically significant. Additionally, we used Pearson correlation analysis to detect the relationships between intestinal bacterial levels, peripheral blood metabolite levels, and PD-related outcomes.

## 3 Results

### 3.1 Aerobic exercise improves motor function and cognitive impairment in MPTP-modeled PD mice

**Figure 1:**
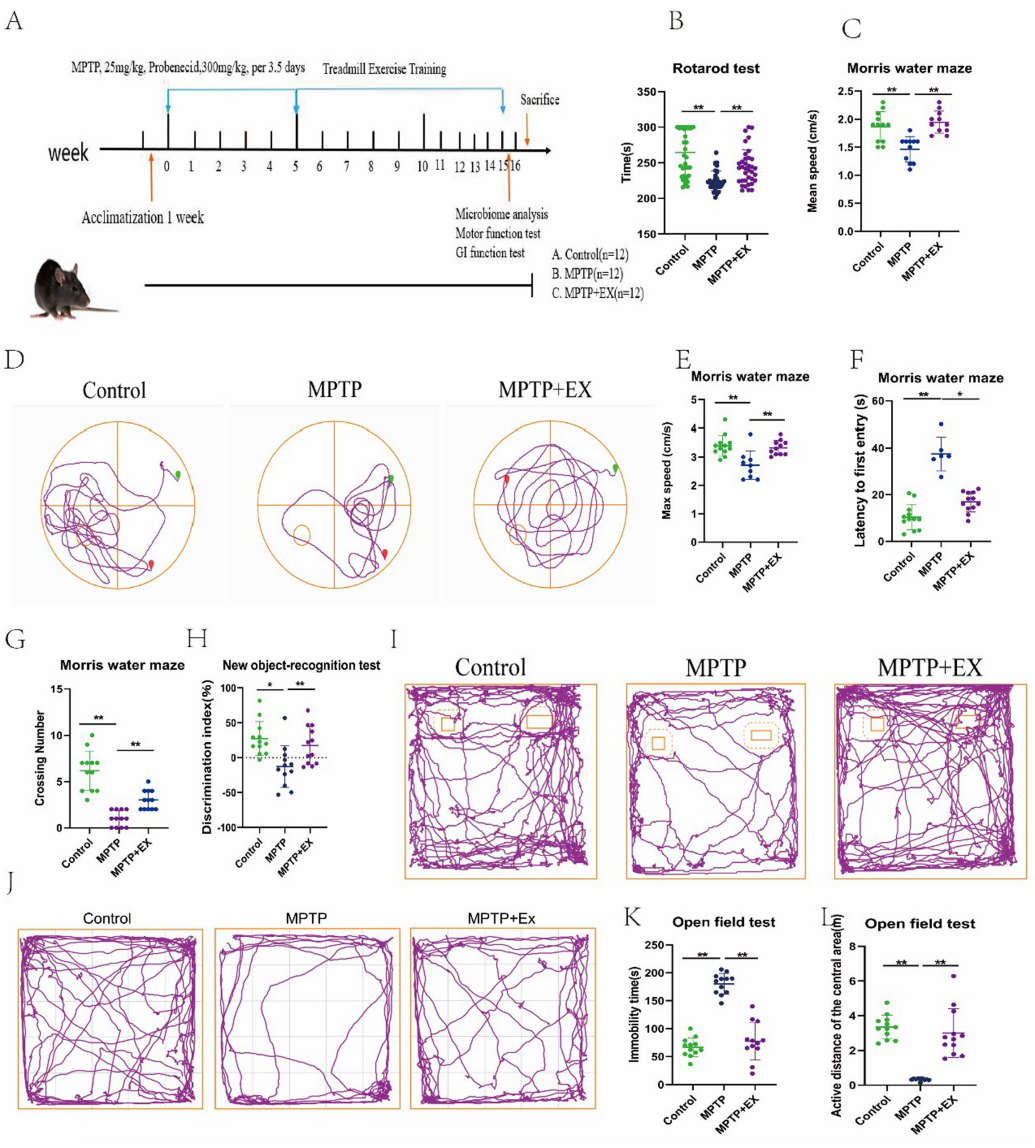
Results of behavioral tests of experimental animals in the first stage. **(A)** Experimental plan; **(B)** pole climbing experiment; **(C–G)** water maze experiment. **(H,I)** New object recognition experiment. **(J–L)** Open field experiment. *n* = 12, *p < 0.05, **p < 0.01.

MPTP is neurotoxic and specifically damages dopamine neurons in the striatum. This loss of dopamine neurons is fundamental to the pathology of Parkinson’s disease (Johansson et al., 2020). To explore how aerobic exercise affects motor and cognitive functions in Parkinson’s disease mice, we used a model. First, we continuously injected MPTP intraperitoneally for 35 days to induce neurotoxicity (Wang et al., 2009). Then, we carried out 8 weeks of aerobic training (Marino et al., 2023; Figure 1A).

We used four behavioral tests to measure the mice’s motor and cognitive functions. In the rotarod test, we measured the latency to fall to evaluate the mice’s muscular endurance. Similar to previous studies (Wang et al., 2024), Parkinson’s disease mice had a shorter drop time than the normal group (Figure 1B). However, mice in the aerobic exercise group had a significantly longer drop time. This shows that muscular endurance was reduced in Parkinson’s disease mice compared to normal ones, and aerobic exercise could improve this deficit.

The water maze test results also support this. The mean and maximum swimming speeds in the water maze were notably higher in the aerobic exercise group than in the Parkinson’s disease group (Figures 1C–E). Changes in swimming speed in the water maze reflect changes in muscular endurance and locomotor ability. So, aerobic exercise can enhance the locomotor ability of Parkinson’s disease mice.

The water maze test can effectively evaluate the learning efficiency and cognitive function of mice (Rafie et al., 2023). In this experiment, Parkinson’s disease mice had a longer latency time and fewer platform crossings compared to the normal group (Figures 1F,G). This indicates that their spatial memory ability was impaired. But in the aerobic exercise group, the latency time shortened, and the number of platform crossings increased. This shows that aerobic exercise can improve the spatial memory cognitive dysfunction in these mice.

To confirm this further, we used the new object recognition experiment. We calculated the recognition index (Figures 1H,I) to assess the mice’s ability to explore new objects and their spatial memory capacity. Similar to previous findings (Liu et al., 2024), the discrimination index of the aerobic exercise group was significantly higher than that of the Parkinson’s disease group. This shows that aerobic exercise can enhance spatial memory capacity and the ability to learn new objects.

Considering the nonmotor symptoms of Parkinson’s disease, depressive mental disorders can significantly affect cognitive function (Wang et al., 2021). So, we did open-field test. The results showed that Parkinson’s disease mice had more resting time (Figures 1J,K) and moved less in the center region (Figure 1L) in the open field compared to normal mice, indicating significant depressive symptoms. However, aerobic exercise mice had less resting time and more movement in the center region compared to Parkinson’s disease mice. This shows that aerobic exercise can improve the depression and mental disorders in Parkinson’s disease mice, which indirectly improves their cognitive function.

### 3.2 Aerobic exercise restores dopaminergic (DA) neuronal damage in MPTP-induced injured mice

Tyrosine hydroxylase (TH) plays a crucial role in the conversion of L-tyrosine to dihydroxyalanine (dopa), the immediate precursor of dopamine. Dopamine is then produced through decarboxylation within dopamine neurons. As the rate-limiting enzyme in this process, TH serves as a reliable marker for dopamine neurons (Zhou et al., 2022). A reduction in TH levels is a clear indication of dopamine neuron loss. Since the loss of DA neurons is a characteristic pathological feature of Parkinson’s disease (PD), we employed immunofluorescence (IF) and western blotting techniques to analyze DA neurons within the nigrostriatal system.

**Figure 2:**
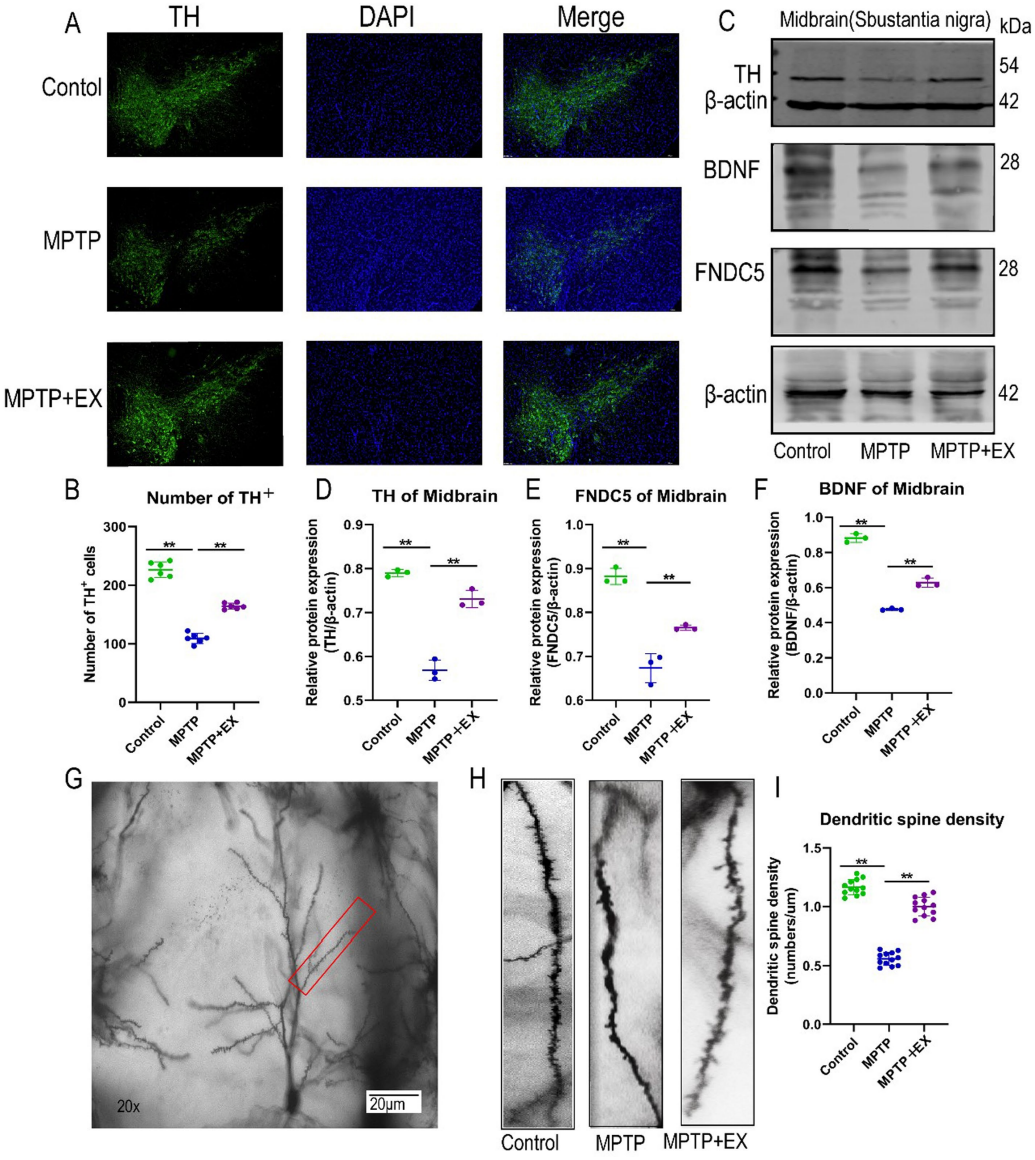
SNpc neuron damage in experimental animals in the first stage. **(A,B)** Midbrain substantia nigra immunofluorescence staining, blue fluorescence for DAPI, green fluorescence for TH + cells. **(C–F)** WB midbrain TH, FNDC5, BDNF, β-actin strip and statistical map. **(G–I)** Hippocampal dendrite spine density and statistical map. *n* = 3, **p < 0.01.

In the IF experiments using the TH antibody, we observed that, in comparison to the normal group, Parkinson’s disease mice had a more substantial loss of dopamine neurons and their projection fibers (Figures 2A,B). However, following aerobic exercise, there was a partial restoration of these reduced neurons. This strongly suggests that aerobic exercise can repair damaged dopamine neurons and their projection fibers in Parkinson’s disease mice.

The western blotting results of TH further validated these findings. The relative TH protein content (TH/internal reference protein) was significantly lower in Parkinson’s disease mice compared to normal mice (Figures 2C,D). Conversely, aerobic exercise led to an increase in the relative TH protein content ratio, which was in line with the results obtained from the IF experiments. Thus, aerobic exercise is shown to restore damaged dopamine neurons and their projection fibers in Parkinson’s disease mice.

### 3.3 Aerobic exercise increases FNDC5-BDNF trophic factor expression and neuronal dendritic spine density in the hippocampal CA1 area of Parkinson’s disease mice

Fibronectin type III structural domain 5 (FNDC5) is a glycosylated type I membrane protein. It is an important regulator in the context of exercise, participating in various metabolic activities and influencing cognitive ability (Belviranlı et al., 2024). However, its role in Parkinson’s disease mice has been relatively under-studied. To address this, we used western blotting to measure the relative content of FNDC5 protein in the hippocampus (FNDC5/Internal Reference Protein).

The results demonstrated that Parkinson’s disease mice with MPTP-induced neurotoxicity had a lower relative FNDC5 protein content compared to normal mice (Figures 2C,E). In contrast, mice in the aerobic exercise group had an elevated relative protein content compared to Parkinson’s disease mice.

To further explore the cognitive improvement and neuroprotective effects of aerobic exercise in Parkinson’s disease mice, we examined the expression of brain-derived neurotrophic factor (BDNF) using western blotting. The relative content of BDNF in the hippocampus of mice in the aerobic exercise group (BDNF/Internal Reference Protein) was significantly higher than that in Parkinson’s disease mice (Figures 2C,F).

In addition, we utilized Golgi staining to observe the density of neuronal dendritic spines in the CA1 region of the hippocampus. Compared with Parkinson’s disease mice, we found that aerobic exercise reversed the damage caused by MPTP to hippocampal neurons (Figures 2G–I). It restored the density of neuronal dendritic spines in the CA1 region of the hippocampus and enhanced the maturity of these spines.

Overall, these results indicate that aerobic exercise increases the expression of FNDC5 and BDNF in the hippocampus and restores the density of dendritic spines in the hippocampal CA1 area. Through these mechanisms, aerobic exercise exerts a neuroprotective effect on Parkinson’s disease mice, thereby improving their memory and cognitive functions.

### 3.4 Aerobic exercise improves gastrointestinal function, enhances intestinal barrier function, and boosts intestinal flora richness

**Figure 3:**
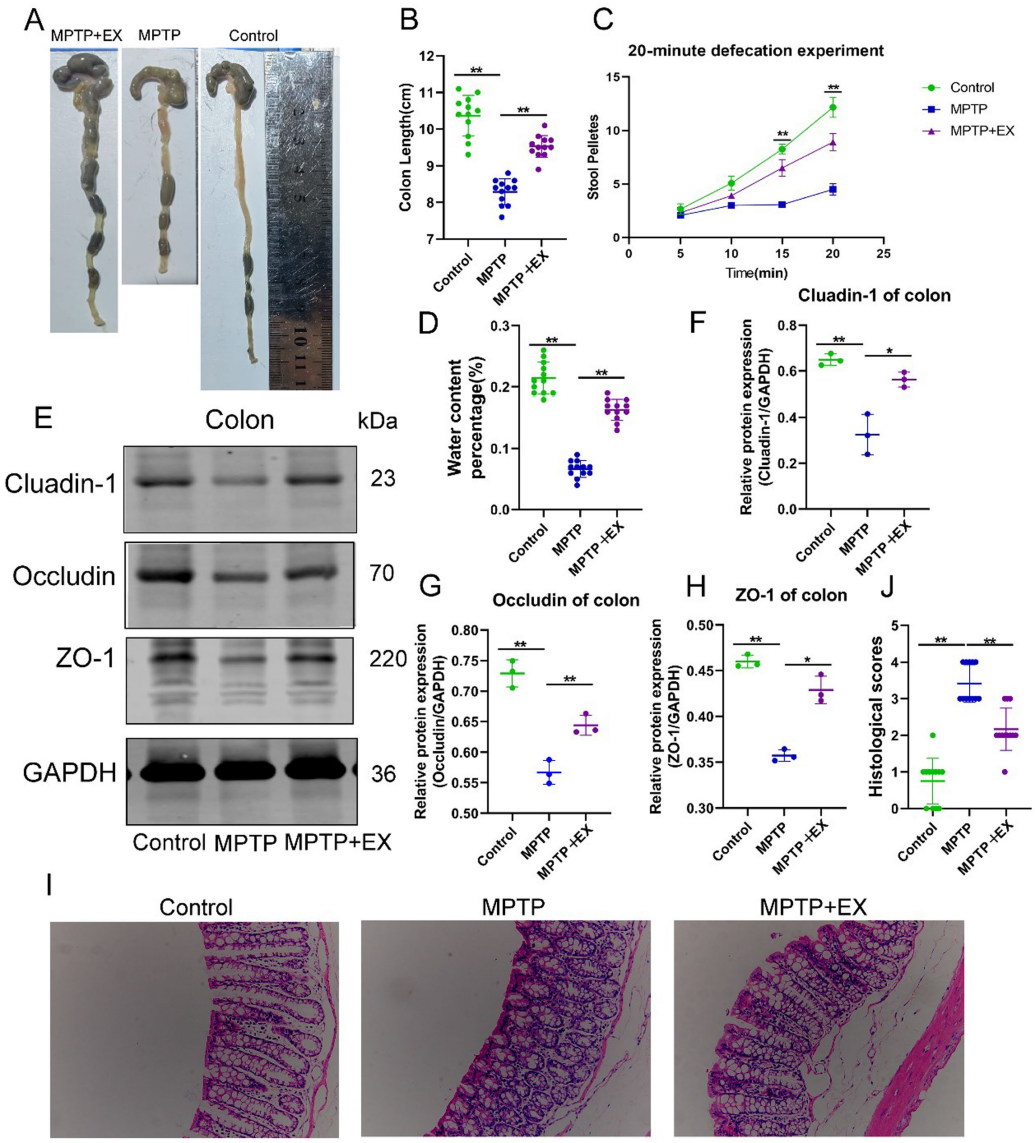
Intestinal function of experimental animals in the first stage. **(A,B)** Colon length; **(C)** 20 min defecation frequency; **(D)** fecal water content, *n* = 12. **(E–H)** Intestinal barrier protein bands and statistical maps; **(I,J)** colon HE staining and histological score. *n* = 3, **p < 0.01.

To assess gastrointestinal function in mice, we measured colon length and defecation frequency. During colon sampling, we discovered that, compared to the normal group (Figures 3A,B), the Parkinson’s disease group, affected by the neurotoxic drug MPTP, had a shorter colon length. In contrast, the aerobic exercise group had a significantly longer colon length compared to the Parkinson’s disease group.

When observing the defecation frequency of mice after 24 h of fasting followed by 2 h of free-feeding, similar trends emerged (Figure 3C). Parkinson’s disease mice had a decreased defecation frequency and lower water content in the cecal pellets compared to the normal group (Figure 3D). In contrast, the aerobic exercise group had an increased defecation frequency and higher water content in the cecal pellets compared to the Parkinson’s disease group. These data suggest that aerobic exercise can ameliorate the gastrointestinal dysfunction induced by MPTP neurotoxic drugs.

Next, we used western blotting to analyze the proteins related to intestinal barrier function, including Cluadin-1 (Figures 3E,F), Occludin (Figures 3E,G), and ZO-1 (Figures 3E,H). All these proteins showed significant differences. The content of intestinal barrier proteins decreased due to MPTP neurotoxic drugs, while it increased after aerobic exercise. Similar results were also evident in HE staining (Figures 3I,J). These data indicate that aerobic exercise can enhance the intestinal barrier function affected by neurotoxic drugs and further clarify that aerobic exercise improves gastrointestinal function by strengthening the intestinal barrier.

**Figure 4:**
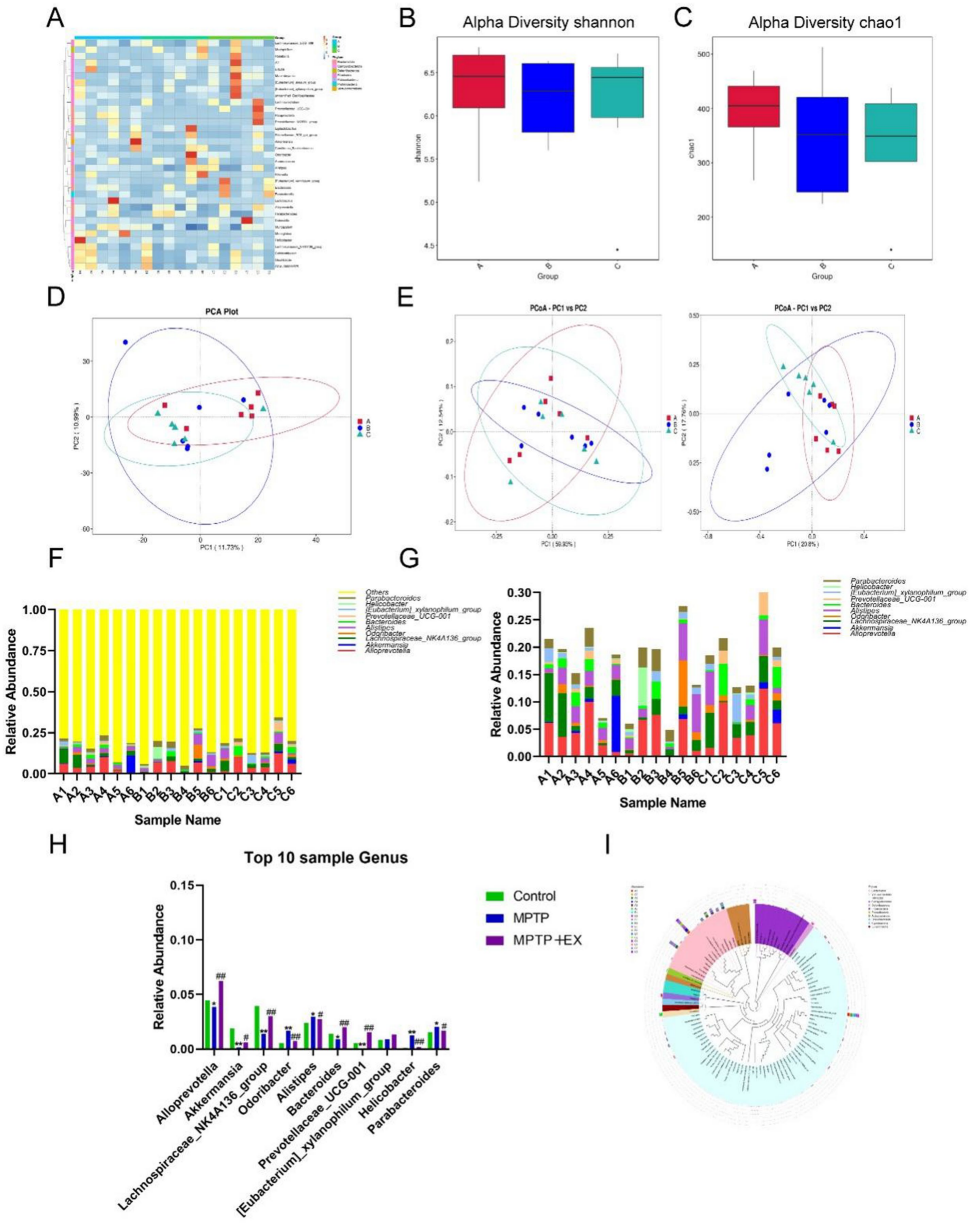
Difference analysis of intestinal flora of experimental animals in the first stage. **(A)** Horizontal heat map of intestinal flora. **(B,C)** α diversity index analysis. **(D,E)** Principal component analysis. **(F–H)** Abundance analysis of different species. **(I)** Species abundance evolutionary tree analysis. Group A was the control group, group B was the Parkinson’s disease mouse group (MPTP group), group C was the Parkinson’s disease mouse aerobic exercise group (MPTP+Ex), *n* = 6, *p was the comparison between group A and group B, and #*p* was the comparison between group B and group C. *p < 0.05, **p < 0.01, #*p* < 0.05, ##*p* < 0.01.

Given that aerobic exercise improves gut function, we used 16S rRNA sequencing to analyze the microbial composition of the gut contents in the three groups. The heat map of the three groups (Figure 4A) revealed that the gut microbiome composition of the Parkinson’s disease mouse group was markedly different from that of the control and aerobic exercise groups. The aerobic exercise group had an increased *α*-diversity and higher values of the Chao1 index and Simpson’s index compared to the Parkinson’s disease mouse group (Figures 4B,C). This indicates that, after 8 weeks of aerobic exercise, the composition of the intestinal flora in the exercise group was significantly distinct from that of the Parkinson’s disease group, with the aerobic exercise training group showing a higher abundance of the gut bacterial community.

Upon further principal component analysis (Figures 4D,E), we found that the principal components of the aerobically trained mice were altered, rectifying the disrupted intestinal flora in Parkinson’s disease mice compared to the Parkinson’s disease mouse group.

To better compare the changes in gut flora after aerobic exercise, we enumerated and compared the top 10 flora at the genus level (Figures 4F–H) and constructed the evolutionary tree of the top 100 species in terms of abundance (Figure 4I). We found that in aerobically trained Parkinson’s disease mice, the intestinal *Alloprevotella, Akkermansia, Lachnospiraceae_NK4A136 _group, Bacteroides, and Prevotellaceae_UCG-001* were more abundant compared to Parkinson’s disease mice, while *Parabacteroides, Helicobacter, Alistipes,* and *Odaribacter* decreased.

In summary, these data suggest that aerobic exercise can significantly improve the composition of host gut microorganisms, reduce the abundance of opportunistic pathogens, increase the species richness of gut flora, and enhance the metabolically active genus levels. 4.5 Intestinal flora mediates neuroprotective effects of aerobic exercise in MPTP-induced Parkinson’s disease mice.

**Figure 5:**
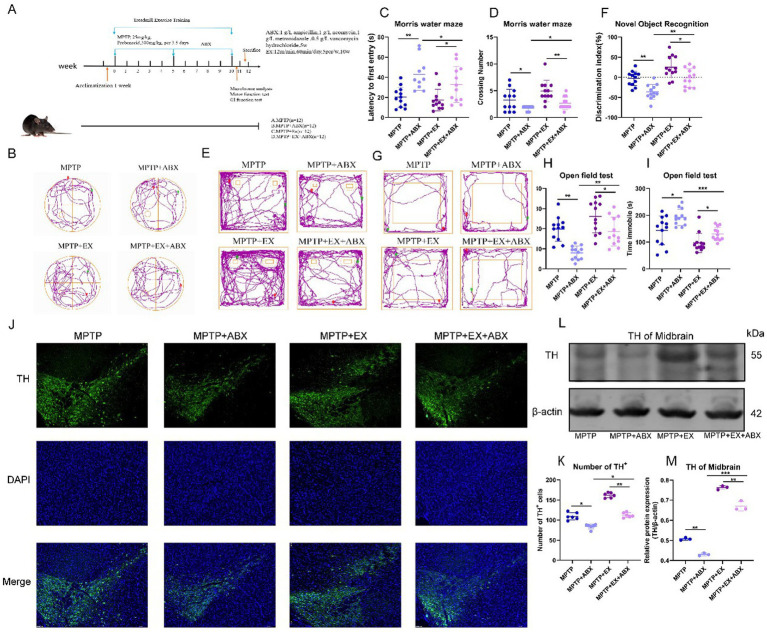
Behavioral tests and SNpc neuron damage in the second stage of animal experiments. **(A)** The second stage animal experiment plan. **(B–D)** Water maze behavioral test. E&F: New object recognition experiment. **(G–I)** open field experiment, *n* = 12. **(J,K)** Midbrain substantia nigra immunofluorescence staining, blue fluorescence for DAPI, green fluorescence for TH + cells. **(L,M)** TH band and statistical map in WB midbrain, *n* = 3. *p < 0.05, **p < 0.01, ***p < 0.001.

We aimed to verify the neuroprotective effects of aerobic exercise mediated by gut flora. We established a new mouse model (Figure 5A). In this model, in addition to using aerobic exercise to modulate gut flora composition in MPTP-induced Parkinson’s disease mice, we introduced an antibiotic mixture to eliminate the gut microbiota. Upon successful establishment of the Parkinsonian model as previously described, MPTP-lesioned mice were randomized into four experimental cohorts (*n* = 12 per group) and subsequently assigned to a 2 × 2 factorial design based on aerobic exercise intervention and antibiotic cocktail administration, as depicted in Figure 5A.

By repeating the previous behavioral experiments, in the water maze experiments, regarding the latency time and the number of platforms crossed (Figures 5B–D), we found that microbial blockade increased the latency time and decreased the number of platforms crossed. Microbial depletion nullified the beneficial effects of aerobic exercise on cognitive improvement in Parkinson’s disease mice. As previously described, to ensure statistical independence across all data samples, each mouse in our experimental design underwent only a single test per experimental paradigm.

Similarly, in the novel object recognition experiment (Figures 5E,F), microbial blockade decreased the discrimination index. In the open-field test, microbe elimination aggravated depressive symptoms in Parkinson’s disease mice. This was manifested as increased resting time and decreased distance traveled in the central region (Figures 5G–I), reducing the beneficial effect of aerobic exercise on improving depressive symptoms in Parkinson’s disease mice.

Next, we explored whether microbial elimination affects the impact of aerobic exercise on DA neuron loss in Parkinson’s disease mice. Immunostaining of TH in the substantia nigra striatum showed (Figures 5J,K) that the reversing effect of aerobic exercise on DA neuronal damage was attenuated by microbial elimination. Microbial elimination led to more DA neuron loss. The western blotting results of striatal TH further confirmed that the elimination of gut flora blocked the neuroprotective effects of aerobic exercise (Figures 5L,M).

The above results indicate that the elimination of microorganisms blocked the neuroprotective effect of aerobic exercise on Parkinson’s disease mice, suggesting that gut flora plays a key role in aerobic exercise-related cognitive function improvement in Parkinson’s disease mice.

### 3.5 Gut flora-mediated aerobic exercise regulates FNDC5-BDNF via PGC1-*α*/CREB

As previously mentioned, FNDC5 and BDNF play important roles in the impact of aerobic exercise on cognitive function in Parkinson’s disease mice. We further investigated whether aerobic-exercise-associated intestinal microbes also regulate the secretion of FNDC5 and BDNF.

**Figure 6:**
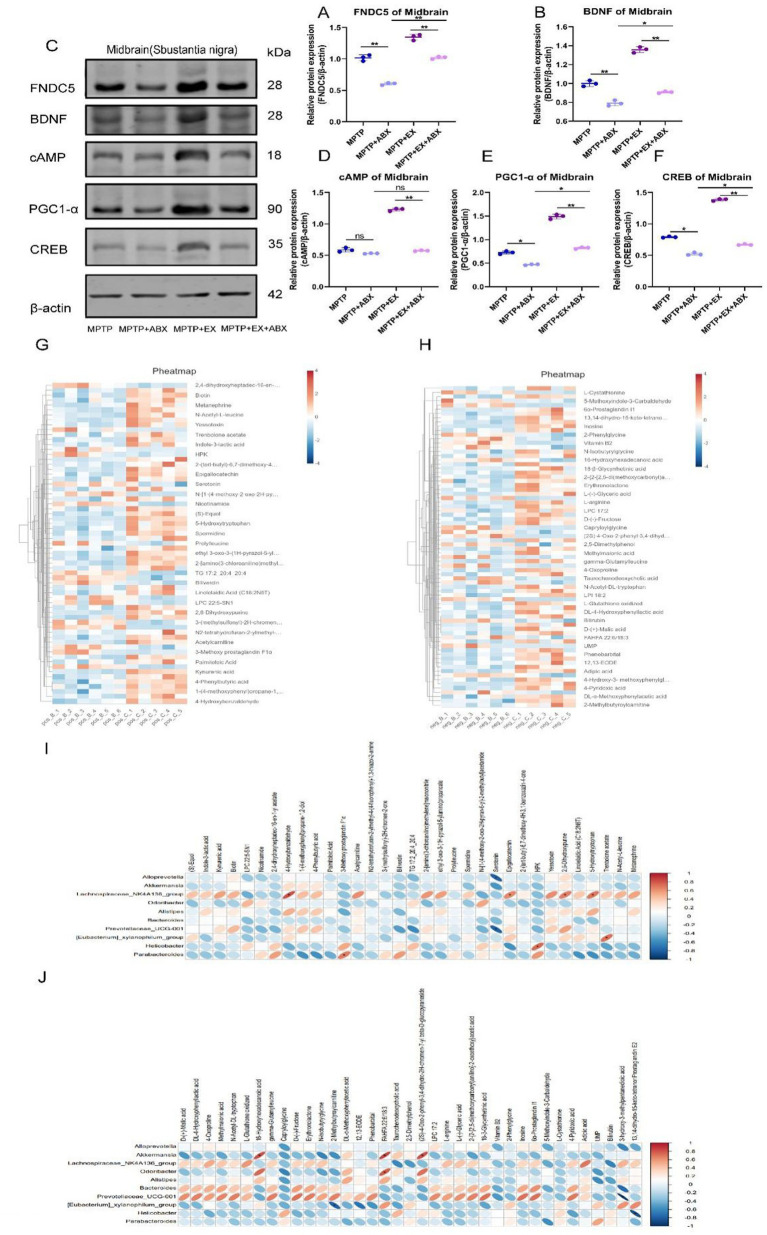
Bands of FNDC5, BDNF, cAMP, PGC1-α, CREB, and β-actin in the midbrain of WB from **A to F** statistical map, *n* = 3. **(G,H)** Clustering heat map of major plasma metabolites. **(I,J)** Heat map of correlation between major intestinal differential bacteria and major plasma metabolites, *n* = 6. Pos: anode, Neg: cathode, Parkinson’s disease mice in group B (MPTP group), aerobic exercise mice in group C (MPTP+Ex group), *p < 0.05, **p < 0.01. This schematic illustrates the pathway through which aerobic exercise mediates cognitive rehabilitation in Parkinson’s disease, based on our investigational results.

Under the above-mentioned model, we used western blotting experiments to examine the relative protein content (FNDC5/internal reference protein, BDNF/internal reference protein) of FNDC5 and BDNF expression in the striatum (Figures 6A–C). The results showed that after microorganism elimination, the expression levels of FNDC5 and BDNF decreased, eliminating the ameliorative effect of aerobic exercise on FNDC5 and BDNF.

In addition, we found that the regulation of FNDC5 by the cAMP/PGC1α/CREB pathway was also influenced by microbes. In further western blotting experiments (Figures 6A,D–F), we discovered that aerobic exercise had a significant activating effect on the cAMP-PGC1-α-CREB pathway in Parkinson’s disease mice. However, after aerobic exercise, microbial elimination inhibited this activating effect.

We found that changes in gut flora affect the activation state of the cAMP-PGC1-α-CREB pathway, thus controlling the secretion of FNDC5. Gut flora mediates the role of aerobic exercise in regulating FNDC5 secretion via PGC1-α/CREB and influences the production of BDNF.

### 3.6 Changes in intestinal flora and correlation of major plasma metabolites

To gain a better understanding of how significant flora alterations and aerobic exercise confer neuroprotection in Parkinson’s disease mice, we conducted untargeted metabolomics sequencing. We collected major plasma metabolites from different groups of mice. The results revealed significant differences in metabolites between anodized and cathodized mice after aerobic exercise compared to Parkinson’s disease mice (Figures 6G,H).

To further clarify the relationship between differential intestinal genera and major plasma metabolites, we performed a correlation analysis between intestinal genera and major plasma metabolites (Figures 6I,J). We found that in the anode group, *Alloprevotella, Akkermansia, Bacteroides, Prevotellacea_UCG-001, Parabacteroides, Helicobacter, Alistipes, Odaribacter* were negatively correlated with most major plasma metabolites, while.

*Lachnospiraceae_NK4A136 _*group was positively correlated with most major plasma metabolites. In the cathode group, *Parabacteroides* and *Helicobacter* were negatively correlated with most major plasma metabolites, and *Bacteroides* and *Prevotellacea_UCG-001* were positively correlated with most major plasma metabolites.

Collectively, these findings further highlight the crucial role of aerobic exercise in remodeling gut microbes and thereby regulating FNDC5. This contributes to our understanding of the mechanisms through which aerobic exercise improves cognitive function in Parkinson’s disease mice.

## 4 Discussion

In this study, we explored the effects of aerobic exercise on Parkinson’s disease mice from the perspective of the gut-brain axis via gut flora. When aerobic exercise is applied to Parkinson’s disease mice, it can enhance intestinal function and the intestinal barrier by improving the composition of the intestinal flora. Additionally, it stimulates the secretion of BDNF, which exerts neuroprotective effects and improves the cognitive function of these mice. Notably, aerobic exercise regulates FNDC5 secretion by remodeling the gut flora. Our data offer new evidence for the role of aerobic exercise in modulating the microbiota–metabolism axis related to PD pathology and provide fresh insights into the pathogenesis of PD.

Previous research has shown that BDNF (brain-derived neurotrophic factor) is crucial. Reduced BDNF expression leads to imbalances in neuronal function, which in turn impacts cognition and memory (Wrann et al., 2013). Aerobic exercise can positively regulate BDNF expression (Kaagman et al., 2024), which aligns with our findings. In Alzheimer’s disease rats, BDNF was found to positively regulate the secretion of FNDC5 by muscle cells (Hegazy et al., 2022), thereby improving cognitive function in these rats.

In our study, we observed that aerobic exercise promotes the expression of FNDC5 in the hippocampal region, consistent with previous reports (Tang et al., 2023), and also enhances the release of BDNF. This modulation increases the synaptic plasticity of hippocampal neurons (Solinas et al., 2019). Our study further demonstrated that this synaptic plasticity reverses the damage caused by neurotoxic drugs to the dendrites of hippocampal neurons, increasing the density and maturity of dendrites. Moreover, aerobic exercise enhances the projection of dopaminergic (DAergic) neurons from the substantia nigra to the hippocampus, an effect that significantly contributes to improving cognitive function. Additionally, previous studies have indicated that the interaction between BDNF and FNDC5 may involve other neuroprotective mechanisms, such as promoting neuroregeneration (Villamil-Parra and Moscoso-Loaiza, 2024), inhibiting neuroinflammation (Qiu et al., 2024; Choi et al., 2024), and slowing apoptosis (Ali et al., 2024). Collectively, these mechanisms hold potential as therapeutic strategies for neurodegenerative diseases.

The etiology of PD remains unclear, but it is highly correlated with age, polygenic mutations, toxic exposures, and brain injury (Kou Li et al., 2018). In recent years, the impact of gut dysbiosis on PD patients has attracted significant attention. Compared to healthy individuals, PD patients have a significantly reduced number of intestinal microbial communities, lower flora abundance, and increased intestinal permeability. These changes may trigger inflammatory responses and central nervous system inflammation (Barichella et al., 2019). It has been reported that the abundance of *Prevotellaceae* and *Lachnospiraceae* (including the genus Roseburia) is significantly decreased in PD patients, while the abundance of *Verrucomicrobiaceae* (including the genus *Akkermansia*) and *Lactobacillaceae* increases (Keshavarzian et al., 2015). The Prevotella family plays a vital role in intestinal protein degradation, intestinal mucus formation, and the production of short-chain fatty acids (SCFAs) through fiber fermentation. A decrease in the abundance of the Prevotella family may lead to reduced intestinal mucus secretion and decreased microbial-derived SCFAs, which could be key factors contributing to increased host intestinal permeability and the acceleration of localized intestinal inflammation (Pietrucci et al., 2019). Furthermore, gut flora dysbiosis accelerates the progression of PD pathology (Bhattarai et al., 2021). The degree of gut flora imbalance varies among PD patients of different ages, and as the disease progresses, the increasingly disrupted gut flora exacerbates their cognitive dysfunction (Jones et al., 2020). Therefore, improving the gut flora of PD patients may present a novel therapeutic strategy for treating PD and its associated cognitive dysfunction.

Exercise therapy is an important part of PD patient rehabilitation (Johansson et al., 2022). Aerobic exercise can improve both motor and non-motor symptoms of patients, enhancing their quality of life (Sacheli et al., 2019). Current research on the underlying mechanisms of aerobic exercise in improving motor symptoms in PD patients mainly focuses on reducing neuroinflammation, promoting the release of neurotrophic factors, enhancing neurogenesis, and increasing DA secretion in the caudate-putamen (Ishaq et al., 2024; Romero Garavito et al., 2024). However, there has been little research on the impact of aerobic exercise on the gut microbiome in PD. In this study, we used MPTP to induce a chronic mouse model of Parkinson’s disease. The PD model led to an imbalance in gut flora, and then aerobic exercise was introduced. Similar to previous studies (Motiani et al., 2020; Akazawa et al., 2023), we found that regular long-term aerobic exercise improved the gut flora imbalance in PD. There was a significant increase in the number of gut microbiota, an enhanced intestinal barrier, improved intestinal function, an increase in the abundance of probiotic bacteria, and a decrease in the abundance of opportunistic pathogenic bacteria. Decreased probiotic abundance and increased opportunistic pathogenic bacteria are considered gut factors that accelerate the pathologic process of PD (Li et al., 2023), which can be reversed by aerobic exercise, consistent with our results. Among them, the abundances of *Alloprevotella, Akkermansia, Lachnospiraceae_NK4A136 _group, Bacteroides, and Prevotellaceae_UCG-001* increased, while those of *Parabacteroides, Helicobacter, Alistipes,* and *Odaribacter* decreased significantly.

To validate the neuroprotective effects of gut-flora-mediated aerobic exercise in Parkinson’s disease mice, we used antibiotics to eliminate gut flora. After aerobic exercise remodeled the gut flora in Parkinson’s disease mice and then the gut flora was eliminated by antibiotic cocktail, the cognitive function improvement, depressive state alleviation, reversal of DA neuron loss, increase in BDNF levels, and activation of the FNDC5 pathway brought about by aerobic exercise were blocked. This suggests that gut flora mediates the neuroprotective effects of aerobic exercise in Parkinson’s disease mice. On the other hand, previous studies have shown that transplantation of curcumin-treated beneficial microbiota into Parkinson’s disease mice (Cui et al., 2022) reversed the loss of DA neurons, alleviated *α*-Syn aggregation, and reduced neuroinflammation in these mice, producing similar neuroprotective effects. Transplanting beneficial bacteria colonies into Parkinson’s disease mice (Sun et al., 2018) could protect these mice through inhibiting neuroinflammation and reducing TLR4/TNF-α signaling. In summary, intestinal flora plays a crucial role in aerobic exercise-induced improvement of cognitive function in Parkinson’s disease mice. Intestinal flora mediates the neuroprotective effect of aerobic exercise in Parkinson’s disease mice, and improving the intestinal flora in PD provides a new therapeutic strategy for treating Parkinson’s disease.

Surprisingly, aerobic exercise in Parkinson’s disease mice significantly activates the cAMP/PGC1α/CREB pathway, and microbial elimination after aerobic exercise inhibits this activation. A similar “blocking effect” of gut microbiome elimination by antibiotics (Cui et al., 2022) coincides with the inhibition of FNDC5 pathway activation. Studies on cognitive dysfunction in the elderly have shown that aging-induced cognitive dysfunction is associated with reduced expression of PGC1-α, FNDC5, and BDNF in the hippocampus, and aerobic exercise can improve cognitive function (Belviranlı and Okudan, 2018). Knocking down FNDC5 in mice revealed an imbalance in the intestinal flora (Liu et al., 2023), suggesting a possible bidirectional pathway between intestinal flora and FNDC5. Our study partially confirms this. In conclusion, there may be bidirectional communication between FNDC5 and the intestinal flora, and the specific mechanism of their interaction requires further investigation.

## 5 Conclusion

Aerobic exercise ameliorates gut flora imbalance and attenuates PD-associated pathological impairments and cognitive deficits in PD mice, but its efficacy on non-motor symptoms can be eliminated by antibiotics, and gut flora-mediated aerobic exercise exerts neuroprotective effects on PD by regulating FNDC5 secretion via PGC1-*α*/CREB.

## Data Availability

The original contributions presented in the study are publicly available. The metabolomics data can be found in the MetaboLights repository here, https://www.ebi.ac.uk/metabolights/MTBLS13123, and the microbial genomics sequencing data have been submitted to the NCBI BioProject database here: https://www.ncbi.nlm.nih.gov/bioproject/PRJNA1344761.

## Footnotes

^1^ https://www.genome.jp/kegg/pathway.html

^2^ https://hmdb.ca/metabolites

^3^ http://www.lipidmaps.org/
